# Unexpected Improvements of Spatial Learning and Memory Abilities in Chronic Rotenone Intoxicated Mice

**DOI:** 10.1371/journal.pone.0091641

**Published:** 2014-03-11

**Authors:** Fengju Jia, Ning Song, Chenyang Zhao, Junxia Xie, Hong Jiang

**Affiliations:** Department of Physiology, Shandong Provincial Key Laboratory of Pathogenesis and Prevention of Neurological Disorders and State Key Disciplines: Physiology, Medical College of Qingdao University, Qingdao, China; University of Lancaster, United Kingdom

## Abstract

The liposoluble insecticide rotenone is commonly used as a mitochondrial complex I inhibitor to replicate Parkinson's disease (PD) pathological features. However, there was no assessment of the spatial learning and memory abilities in chronic rotenone-induced PD models. In the present study, by rotarod test and Thioflavine T staining, we first noted the impairment of motor coordination in rotenone-treated group for 3 months, as well as alpha-synuclein inclusions in the nigral dopaminergic neurons in C57BL/6 mice with intragastrical delivery of rotenone (5 mg/Kg) for 3 months rather than 1 month. We then evaluated spatial learning and memory abilities by Morris water maze task in this model. The results showed escape latency reduced in rotenone-intoxicated mice for 3 months, indicating an improvement of learning ability. However, it was delayed slightly but not significantly in rotenone-intoxicated mice for 1 month. Similarly, we demonstrated that spatial memory ability was enhanced in 3-month-treatment group, but impaired in 1-month-treatment group. There were no proliferating cell nuclear antigen and doublecortin positive cells in the hippocampus by double immunofluorescent staining, indicating the absence of hippocampal neurogenesis in rotenone-intoxicated mice. These results suggest that spatial learning and memory abilities are disturbed in chronic rotenone-intoxicated PD model.

## Introduction

Parkinson's disease (PD), as the second most prevalent neurodegenerative disease, affects as many as 1–2% of the worldwide population over 60 [1]–[3]. It is characterized by selective loss of the dopaminergic neurons in the substantia nigra pars compacta (SN_pc_), a profound loss of dopamine (DA) in the striatum, and the presence of intracytoplasmic inclusions called Lewy bodies (LB) in the remaining dopaminergic neurons [4]–[6]. The cardinal features of PD are tremor, rigidity, bradykinesia, and postural instability. However, there are some nonmotor symptoms, such as insomnia, constipation, cognitive decline, appearing before or in parallel with motor deficits [7]–[10]. A high rate as 84% in cognitive decline was reported in one long-term (15–18 years) follow-up study of PD patients [11].

There is growing evidence that environmental toxicants, such as pesticides, can be associated with an increased risk of PD [12]–[17]. Rotenone, which is commonly used as a liposoluble insecticide, is widely believed to be a high-affinity, specific inhibitor of mitochondrial complex I [18], [19]. Rotenone-induced PD model seems to replicate almost all of the hallmarks of PD including alpha-synuclein aggregation, LB formation and nigral dopaminergic neurons loss [20]. In the present study, we aimed to evaluate changes of spatial learning and memory abilities in chronic PD models by employing an intragastrical model of rotenone intoxication for 1 or 3 months.

## Materials and Methods

### Chemicals and drugs

Unless otherwise stated, all chemicals were purchased from Sigma Chemical Co. (St. Louis. Mo, USA). The primary antibody against proliferating cell nuclear antigen (PCNA) was purchased from Cell Signaling Technology (CST, UK). Antibody against doublecortin (DCX) was from Santa Cruz Biotechnology Inc (CA, USA) and antibody against tyrosine hydroxylase (TH) was from Millipore (Billerica, MA). The Alexa Fluor^®^ 555 donkey anti-mouse IgG, Alexa Fluor^®^ 555 donkey anti-rabbit IgG and Alexa Fluor^®^ 488 donkey anti-goat IgG were from Invitrogen (USA). All other chemicals and reagents were of the highest grade available from local commercial sources.

### Ethics statement

This study was carried out in strict accordance with the recommendations in the Guide for the Care and Use of Laboratory Animals of the National Institutes of Health. The protocol was approved by the Committee on the Ethics of Animal Experiments of Qingdao University. All efforts were made to minimize animal suffering.

### Animal treatment

One-year old C57BL/6 mice were housed at room temperature under a 12-h light/dark cycle. Food and water were delivered ad libitum. Mice were divided into four groups: rotenone-treated groups with 5 days one week administration consecutively for 1 or 3 months and corresponding vehicle-treated groups. In rotenone-treated groups, mice were intragastrically administrated 0.01 mL per animal weight of rotenone solution (0.625 mg/mL rotenone, 4% carboxymethylcellulose and 1.25% chloroform). The vehicle treatment mice received only the vehicle (4% carboxymethylcellulose and 1.25% chloroform) [21].

### Rotarod test

Motor coordination and balance were assessed using a rotarod apparatus (Med Associates, USA). The animals were placed on the rolling rod with an initial speed of 4 rpm. Then, two trials with an interval trial time of one hour were performed using an accelerating speed levels (4 to 40 rpm) mode of the apparatus. The mean latency to fall off the rotarod was recorded.

### TH immunofluorescence and Thioflavine T (ThT) staining

Animals were anesthetized with 8% chloral hydrate (400 mg/Kg, i.p.). Mice were sacrificed with perfusion with 0.9% saline followed by 4% paraformaldehyde. Brains were removed and postfixed in 4% paraformaldehyde for 6 hours, then, transfered to 30% sucrose until sectioning. Sections (20 μm) were cut on a freezing microtome (Leica, Germany). Alternate SN_pc_ sections were double-stained for TH and ThT. After three washes in 0.1 mol/L phosphate buffered saline (PBS; pH 7.4) plus 0.3% Triton X-100, sections were incubated overnight with primary antibody of TH (1∶2000) at 4 °C. Then washed three times with PBS and incubated in the second antibody of Alexa Fluor^®^ 555 donkey anti-rabbit IgG for 2 h at room temperature. Next, sections were rinsed with PBS for three times and incubated in 0.05% ThT solution for 10 min, subsequently differentiated in 80% ethanol for 5 min. The slides were rinsed in distilled water, mounted in 70% glycerin, and stored in a dark chamber.

### Morris water maze test

The Morris water maze consisted of four parts: circular pool (120 cm in diameter), platform, the temperature control system and the trajectory tracking and analysis system. The circular pool was filled with water that had been made opaque by adding a white pigment and was divided into four quadrants: adjacent left (adj.L), training, adjacent right (adj.R) and opposite (oppo). An escape platform (10 cm in diameter) was submerged about 0.5 cm below the water surface, in a fixed position in the training quadrant.

After 1 or 3-months rotenone or vehicle treatment, the mice were first handled for 3 minutes per day for 7 consecutive days. Then, each animal underwent two sessions: training session and probe session. For training session, the mice were subjected to six training trials per day for 7 consecutive days. One average data was collected daily for each animal and totally recorded for 7 consecutive days labeled as day 1 to day 7. The animals were allowed to rest 30 sec on the platform between trials. The system automatically recorded time as escape latency when animals climbed up to the platform.

The spatial memory was assessed with a probe test on day 5 and day 7 of training trials. The platform was removed and the mouse was placed in the opposite quadrant and allowed to search for 60 sec in the pool. Relative time (%) spent in all the four quadrants that is, adj.L, training, adj.R and oppo was recorded. The other parameters of the probe trial were: time to destination (sec), distance travelled (cm), average speed (cm/sec), average proximity (cm), platform crossings. Behavioral data from the training and the probe trials were analyzed using an automated tracking system (Actimetrics, USA).

### Immunofluorescence staining

Alternate hippocampus sections were stained for PCNA and DCX. After three washes in PBS plus 0.3% Triton X-100, sections were incubated overnight with primary antibody of PCNA (1∶1000) and DCX (1∶1000) at 4 °C. Then washed three times with PBS and incubated in the second antibody of Alexa Fluor^®^ 555 donkey anti-mouse IgG and Alexa Fluor^®^ 488 donkey anti-goat IgG for 3 h at room temperature. Next, sections were rinsed with PBS for three times. After staining the nuclei with Hoechst 33258, sections were mounted with 70% glycerin and examined using a Fluorescence Microscope (Carl Zeiss, Germany).

### Statistical analysis

All data were expressed as mean values ± S.E.M. Independent sample t-test was adopted to analyze the rotarod test and parameters at the probe session between the vehicle and rotenone-treated groups (SPSS 19.0 software, n≥9 for each group). The escape latency during the training tests was determined by repeated measure ANOVA analysis (SPSS 19.0 software, n≥10 for each group. *P*<0.05 was considered to be statistically significant.

## Results

### Lesion of motor coordination ability in rotenone-intoxicated mice

**Figure 1:**
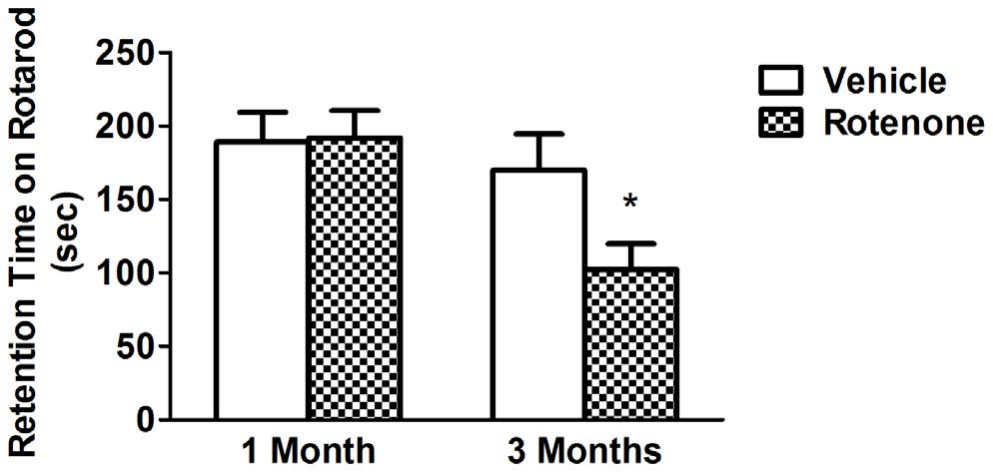
Effects of rotenone on motor coordination ability by rotarod test. Motor coordination ability was assessed by the rotarod test. The residence time of mouse on rotarod treadmills was shorter in mice with rotenone administration for 3 months. There were no variations observed in 1–month-rotenone-treated group compared with the vehicle group.* *P*<0.05, compared with the vehicle; n≥9.

Rotarod test was used to evaluate motor coordination of the rotenone- intoxicated mice. By performing this test, we found that the residence time on rotarod treadmills was shorter in 3-month-treated group, whereas there was no change in 1-month-treated group (Figure 1). These results indicated that motor coordination ability was impaired in mice with intragastrical rotenone administration for 3 months.

### Alpha-synuclein aggregation in the SNPC of rotenone-intoxicated mice

**Figure 2:**
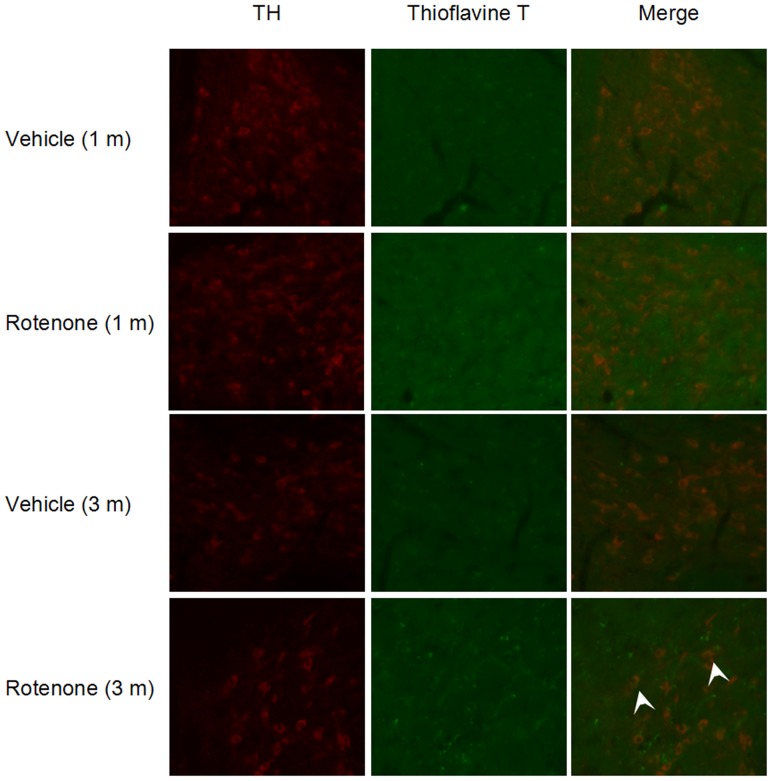
Effects of rotenone on alpha-synuclein inclusion formation in the SN_pc_. Immunostaining against TH (red) and Thioflavine T (green) were evaluated by fluorescence Microscope. Alpha-synuclein inclusions (Thioflavine T positive) were observed in the TH^+^ neurons of the SN_pc_ in mice with rotenone administration for 3 months. No alpha-synuclein aggregations were detected in mice with rotenone administration for 1 month. (Magnification: 400x).

ThT staining was employed to verify whether rotenone could induce alpha-synuclein aggregation in the SN_pc_. We applied ThT to label alpha-synuclein inclusions in different groups. ThT, a dye recognizing aggregates with a β-pleated sheet structure, was widely used for the analysis of alpha-synuclein aggregates [22]–[25]. The results indicated that despite the absence of obvious alpha-synuclein aggregation in the SN_pc_ of 1- month-treated mice, positive ThT staining was detected in the TH^+^ neurons in the SN_pc_ of 3-month-treated mice, indicating intragastrical rotenone administration for 3 months induced alpha-synuclein inclusions in the dopaminergic neurons (Figure 2).

### Effects of rotenone on spatial learning ability at the training session

**Figure 3:**
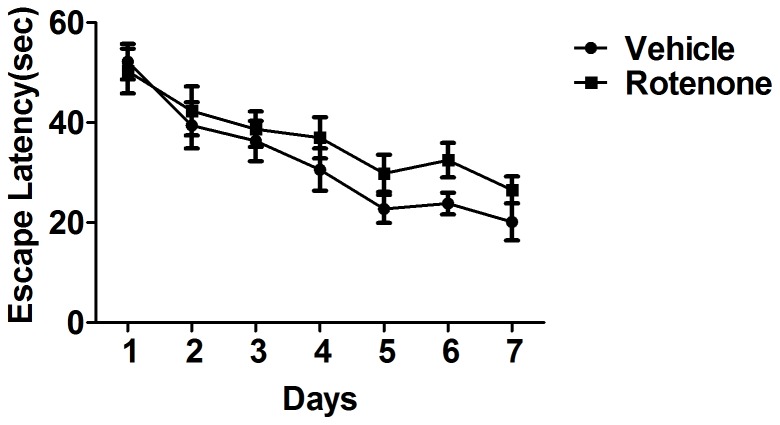
Effects of rotenone for 1 month administration on escape latency at the training session. Although there was a tendency of the latency to escape to be delayed in mice with rotenone administration for 1 month, no significant difference was observed compared with the vehicle. n = 13.

**Figure 4:**
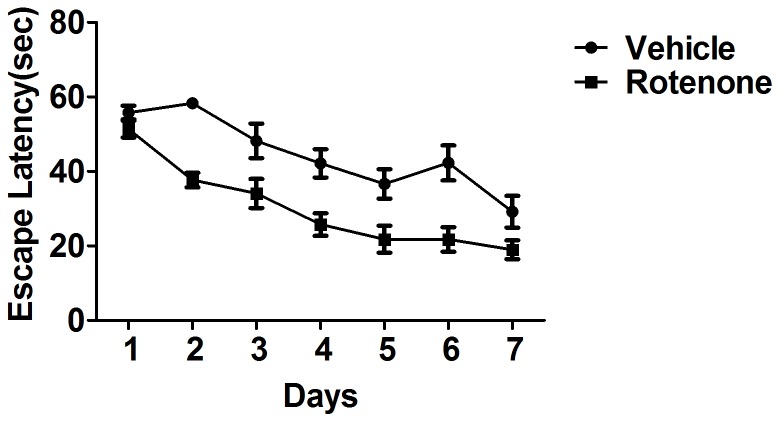
Effects of rotenone for 3 months administration on escape latency at the training session. The latency to escape on the hidden platform reduced in mice with rotenone administration for 3 months. *P*<0.05, compared with the vehicle; n≥10.

At the training session, no gross differences were observed in the latency to escape on the hidden platform between rotenone-intoxicated mice for 1 month and the vehicle, although there was a tendency of the latency to escape to be delayed (Figure 3). Intriguingly, the latency to escape on the hidden platform reduced in rotenone-intoxicated-mice for 3 months (Figure 4).

### Effects of rotenone on spatial memory ability at the probe session

**Figure 5:**
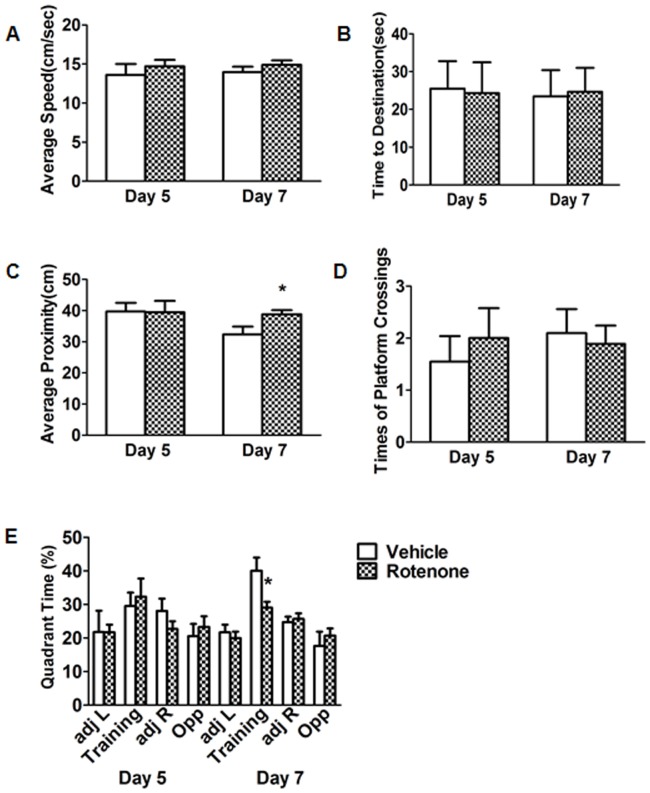
Effects of rotenone for 1 month administration on the parameters of the probe session. On the 5th day no changes were detected in any of the parameters: average speed (A), time to destination (B), average proximity (C), platform crossings (D), relative time in the training and opposite quadrant (E). Nevertheless, average aroximity (C) elevated and relative time spent in the training quadrant (E) decreased on the 7th day. *P<0.05, compared with the vehicle; n = 13.

No changes were detected in 1-month-treated group in any of the parameters of the probe session on the fifth day (Figure 5A-E). Average proximity elevated 19.8% (Figure 5C), nevertheless, relative time spent in the training quadrant decreased 26.4% (Figure 5E) on the seventh day. This indicated a deficit of spatial memory ability in rotenone-intoxicated-mice for 1 month.

**Figure 6:**
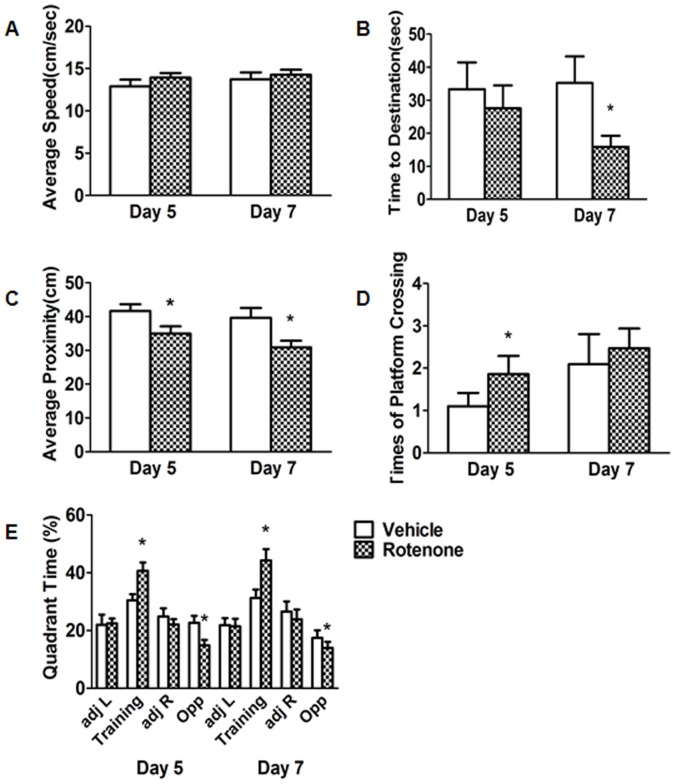
Effects of rotenone for 3 months administration on the parameters of the probe session. A: average speed. There were no variations either on the 5th or the 7th day. B: Time to destination reduced on the 7th day but not the 5th day. C: Average proximity ameliorated both on the 5th and the 7th day. D: Platform crossings improved on the 5th day. E: Quadrant time of training improved in the training whereas declined in the opposite. *P<0.05, compared with the vehicle; n≥10.

In rotenone-intoxicated mice for 3 months, we observed times of platform crossings and relative time in the training quadrant ameliorated 144% and 33.4%, respectively (Figure 6D and E), nonetheless, average proximity and relative time in the opposite quadrant declined 16.0% and 34.5%, respectively (Figure 6C and E) on the fifth day. There was no variations in the other parameters of the probe trial, for instance, average speed and time to destination (Figure 6A and B). However, time to destination, average proximity, and relative time spent in the opposite quadrant reduced 54.9%, 22.2% and 37.3%, respectively (Figure 6B, C and E) on the seventh day, whereas relative time spent in the training quadrant improved 41.4% (Figure 6E). These data suggested that intragastrical administration of rotenone for 3 months enhanced spatial memory ability in mice.

### No obvious neurogenesis was observed in chronic rotenone-intoxicated mice

**Figure 7:**
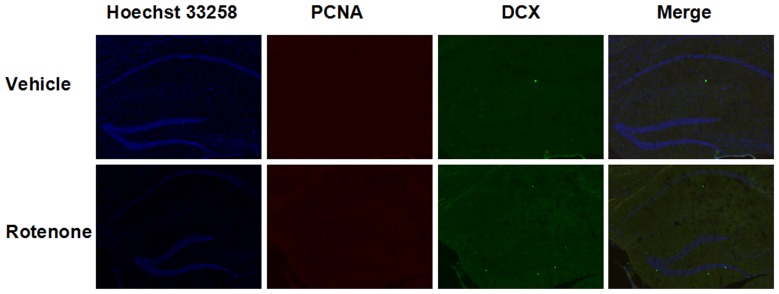
Effects of rotenone for 3 months administration on neurogenesis in hippocampus. Double-immunostaining against PCNA (red) and DCX (green) were labeled for the marker of adult neurogenesis in hippocampus. The blue signal (Hoechst 33358) stained the nuclei. No significant difference was detected between the vehicle and rotenone treated groups. (Magnification: 100x).

To investigate whether the improved spatial learning and memory abilities were related to the hippocampal neurogenesis, we labeled PCNA positive cells and DCX positive cells in hippocampus by double immunofluorescent staining. Nevertheless, no obvious difference was observed in the hippocampus between the vehicle and rotenone-treated groups, indicating the lack of neurogenesis in this region (Figure 7).

## Discussion

In the present study, we adopted a low dose rotenone application as a PD model. We noted the impairment of motor coordination in rotenone-treated-group for 3 months, as well as alpha-synuclein inclusions in the dopaminergic neurons of the SN_pc_. These results suggested chronic intragastrical administrated rotenone replicated successfully the classical features of PD in the present study, which conformed to other reports [26], [27]. Rotenone can induce gastrointestinal tract dysfunction as determined by alpha-synuclein aggregation in the enteric nervous system (ENS), loss of myenteric neurons of the small intestine [28], a delay in gastric emptying, and impaired functioning of inhibitory neurons in the ENS [29]. The local effect of ingested rotenone (orally administered) on the intestinal track could induce alpha-synuclein aggregation in the ENS and thereby induce PD-like pathological progression predicted by Braak's model [30], [31]. This further highlighted the advantage of chronic intragastrical administrated rotenone model.

However, it is largely unknown how spatial learning and memory abilities changes in rotenone-intoxicated model. The present study showed that no changes were observed in spatial learning ability in 1-month-treated group. Similarly, average speed did not vary significantly from rotenone-treated group to the vehicle in probe session, indicating little damage of motor coordination ability. However, a deficit of spatial memory ability was detected in 1- month-treated group. Since it was well accepted that the hippocampus was essential for spatial learning and memory performance on Morris water maze task [32], [33], our results indicated that the popular insecticide rotenone could induce hippocampus damage and thus memory disturbance. This further replicated the findings memory impairment was found in PD patients [34]–[36].

Interestingly, we found that both spatial learning and memory abilities were improved in mice with intragastrical rotenone treatment for 3 months. Presumably, spatial learning and memory abilities are related to hippocampal neurogenesis. However, there seemed no evidence for hippocampal neurogenesis in rotenone-intoxicated mice. Considering the aging hippocampus showed a sharp decline in neurogensis in 10-month to 1-year old mice [37], it is reasonable for hardly detectable neurogenesis in the aging mice used in the present. Studies have also revealed that neurogenesis is required for some but not all hippocampus-dependent tasks [37]–[39]. Adult neurogenesis and memory processing in the hippocampus might not be correlated [40]. In recent years, an increasingly detailed picture of gastrointestinal tract dysfunction, such as abnormal salivation, dysphagia, delayed gastric emptying, constipation, and defecatory dysfunction, has emerged in the setting of PD [41]–[43]. Impaired gastrointestinal tract motility was due to multifactorial but unproven mechanisms, however, it is likely several peptide hormones, for example insulin, leptin and ghrelin, might be involved in this process [44]. These peptides have been reported to have effects on learning and memory [40], [45]–[47]. Therefore, we supposed that peptide hormones might play correlative roles in modulating memory in this chronic rotenone intoxicated models. Further work needs to be done in the near future.

In conclusion, we presented spatial memory abilities disturbance in chronic rotenone-intoxicated PD model. Unexpected improvements in 3-month-treated mice need to be further investigated. This might draw more attention to the learning and memory problems in PD.
